# Explicit and implicit affective attitudes of female athletes towards different body sizes

**DOI:** 10.1186/s40359-025-02567-6

**Published:** 2025-03-14

**Authors:** Petra Jansen, Jelena Haugg, Franziska Anna Schroter

**Affiliations:** Faculty of Human Science, University of Regensburg, Universitätstraße 31, 93053 Regensburg, Germany

**Keywords:** Body image, Female athletes, Lean and non-lean sports, Explicit and implicit attitudes

## Abstract

The present cross-sectional study is the first to investigate the relationship between implicit and explicit attitudes toward different body sizes in female athletes of different sport types. Second, it was examined if these attitudes are, among other factors, related to body satisfaction in these women. In total, 146 female athletes between 18 and 65 from lean and non-lean sports participated in the study, measuring explicit and implicit affective attitudes using pictures of women with different body sizes. Eating disorder risk, the assessment of the image of the own body, and self-compassion were also asked. Results showed that female athletes had more positive explicit affective attitudes towards pictures of individuals whose body sizes correspond to lower body mass indices ranges than higher ones. No differences were found for implicit attitudes. Affective explicit and implicit attitudes for different body sizes showed no significant difference between lean and non-lean sports athletes. Explicit and implicit attitudes did not predict body image satisfaction, but the actual weight-ideal discrepancy, self-compassion, and risk of eating disorders did. Due to the high relevance of body image satisfaction for society and health, future studies should address the aspects of self-compassion and the risk of eating disorders in more detail.

**Supplementary Information:**

The online version contains supplementary material available at 10.1186/s40359-025-02567-6.

## Introduction

This study will investigate whether implicit and explicit attitudes towards various body size pictures are different in female athletes from lean sports compared to non-lean sports and, secondly, whether they are, among other factors, related to body image satisfaction. Lean sports were defined as sports in which leanness and/or low weight were considered necessary, and non-lean sports were defined as sports where these factors are considered less critical [1]. Body image is an umbrella term that includes several constructs and refers to a stable, conscious image of one’s body [2]. It is a complex construct that includes cognitive-affective, perceptual, and behavioral domains. Body image satisfaction and image dissatisfaction reflect the cognitive-affective aspect [3]. Body image satisfaction is given when the perceived actual body image and the ideal body image are in accordance. The actual-ideal weight discrepancy can measure it [4]. Previous research has primarily focused on body image dissatisfaction [5, 6], which can be conceptualized as an over-evaluation of weight and shape concerning a person’s sense of the self [3]. Women seem to have higher scores in dissatisfaction across the lifespan than men [3, 7]. Current research has shifted its focus to relatively positive aspects. Positive body image is characterized by affection and acceptance towards one’s body [8].

### The relation of attitudes towards body size pictures

Attitudes can be categorized into explicit and implicit forms. According to dual-process models, human attitudes are believed to involve both conscious (controlled) and unconscious (uncontrolled) elements [9]. The controlled aspects can be assessed using explicit measures, and the uncontrolled aspects can be assessed using implicit tasks. Recent research suggests that an individual’s explicit attitude may only sometimes correspond to their implicit attitude [10].

Regarding the relation of implicit and explicit attitudes towards pictures of different body sizes in non-athletes, it has been shown that there was a positive effect in watching pictures of women whose body size corresponds to a low BMI, whereas seeing pictures of women whose BMI corresponds to a high BMI had a negative effect on implicit attitudes [11]. The same pattern could be shown in explicit measurements of female Spanish college students [12].

The relation between implicit attitudes toward the concepts of lower and higher BMI ranges and body image satisfaction has been investigated, until now, to the best of our knowledge in four studies with non-athletes: First, implicit desire to be thin tended to be higher in participants who were implicitly more concerned with their body image [13]. Second, Hernández-López [14] showed that Spanish women with low and high body dissatisfaction had implicit positive attitudes toward the concept of a lower BMI. Still, only those with low body image dissatisfaction showed implicit positive attitudes toward a higher BMI. Third, women who were more dissatisfied with their bodies attended to words, which are related to lower and higher BMI more than body-satisfied women [15]. In a study with healthy young women, it was recently shown that there is an explicit positive affective attitude towards images of individuals whose body size corresponds to a lower BMI but not to a BMI of emaciation, whereas a negative bias exists towards images of body sizes in extremely low or extremely high BMI categories [16]. In this study, no difference between categories could be shown when measuring the implicit attitudes. The study also demonstrated that implicit and explicit affective attitudes towards different body sizes are unrelated to the participants` body image satisfaction.

Because athletes spend most of their time on sports [17] and receive a premium on excellence and achievement [18, 19, 20], physical activity and the body play an essential role in their lives. They are concerned with their bodies regarding their physical fitness, level of strength, and, e.g., muscularity. Regarding body image, athletes represent a distinct category within a broader spectrum of individuals.

### Body image in athletes

Different factors can negatively influence athlete body image satisfaction, such as social pressure from coaches or performance-related factors, such as a specific weight requirement for their sport [21, 22]. Additional factors for concerns in body image satisfaction could be the background of sports training, the intensity of training or training regime, sports uniforms, and regular weight measurements [23, 24, 25, 26, 27, 28]. On the other hand, body image satisfaction can also be promoted through sports, as athletes appreciate their bodies for what they can achieve [29]. Intervention studies have also shown that exercise can improve body image compared to non-exercising control groups [25].

Regarding the body image concerns of athletes of various sports, the results differ depending on the participant’s sex: It has been shown that only men but not women in lean sports reported more body image dissatisfaction than in non-lean sports [30]. In a systematic review, Varnes et al. [31] use another sports type differentiation and show that athletes in sports that are frequently perceived as being intended more for women (e.g., gymnastics, volleyball, and tennis) have a greater risk for body image concerns than athletes in endurance sports (swimming, long-distance running, and water polo). Also, athletes who practice their sport at a higher competition level were less satisfied with their bodies [31], indicating a negative relation between body image satisfaction and division level.

### Negative and positive factors related to body image

Moreover, body image in athletes is not only related to the type of sport and the competition level but also to negative and positive factors. One often-mentioned negative factor is the eating disorder risk [32], which is in line with the relation of general concerns about body image and eating disorders [33, 34]. In the study of Whitehead et al., [32] eating disorders in the form of pathogenic weight control measures (PWCM) were related to body dissatisfaction. However, in another study, the eating disorder risk in women did not differ between women who practiced a lean or a non-lean sport [35]. This contradicts a study by Kong and Harris [34], which showed higher body image dissatisfaction and eating concerns in female athletes practicing a lean sport, regardless of participation level. The results align with the results from the review of Chapa et al. [36], showing that female lean-athletes were at higher risk for eating concerns, drive for thinness, restricting, and loss-of-control eating compared to non-lean sports relative to non-athletes.

Next to the risk factors related to body image satisfaction, protective factors such as self-compassion affect body image more positively [37]. Self-compassion is seeing oneself as a good friend [38]. It can be distinguished into positive scales of self-kindness, common humanity, and mindfulness and the negative scales of self-judgment, isolation and over-identification [39]. Self-compassion was positively related to body satisfaction in female university students [40]. Whether self-compassion differs between athletes of different types of sports must be questioned. One study showed positive relations of self-compassion and mindfulness on body image satisfaction [16]. This study investigated attitudes toward different body sizes but did not investigate whether participants came from lean or non-lean sports backgrounds.

### Main goal of the study

This study investigates the explicit and implicit affective attitudes toward pictures of women whose body size corresponds to different BMI ranges in a sample of female athletes from lean and non-lean sports. Furthermore, it will be investigated how those attitudes are related to body image satisfaction in female athletes of lean and non-lean sports. Relating factors, possibly negative, such as the risk of an eating disorder and positive, such as self-compassion, are included. In detail, the following hypotheses can be formulated:

a) A positive explicit and implicit affective attitude towards pictures of women whose body size corresponds to lower BMI ranges and a negative attitude towards pictures of individuals whose body size corresponds to higher BMI ranges are expected.
  b) It is hypothesized that female athletes in lean sports show a higher positive affective attitude toward pictures of individuals whose body size corresponds to the lowest BMI ranges than athletes in non-lean sports.
  c) Within the group of female athletes who practice a lean sport, the pictures of individuals whose body size corresponds to the lower and average BMI ranges is expected to have a better affective rating than the pictures showing women whose body size corresponds to the higher two BMI categories. For non-lean athletes, it was anticipated that the explicit affective ratings of female pictures whose body size corresponds to the lower, average, and higher BMI ranges should be better than those of non-lean athletes.
Self-compassion is anticipated to positively predict body image satisfaction, whereas eating disorder risk may negatively predict it. Due to athletes’ preoccupation with their bodies, implicit and explicit attitudes toward body size corresponding to the lowest BMI ranges are expected to predict it, as well as a possible interaction between group and explicit and implicit attitudes. BMI and body image satisfaction, namely the actual-ideal weight discrepancy, will be included as assumed predicting factors because both significantly predict body image satisfaction in a former with a non-athlete sample [16].

## Methods

Participants in this cross-sectional study were female athletes from lean and non-lean sports in south Germany. They were asked to participate in an online experiment via newsletter, social media, or personal recruitment. The experiment was conducted by the participants on a laptop or computer. As compensation, participants were given either six Euros or course credit if they were students. The experiment could be terminated at any time. Based on the results of Roddy et al. [11], a medium effect size for the difference between the implicit and explicit ratings of the different body sizes depending on the group (lean and non-lean sports) was expected. Inclusion criteria were age above 18 years and practice of the respective sport for at least eight years, at least twice a week, according to the studies of Jansen et al. [41, 42]

Three hundred sixty-seven participants started the online study by clicking on the study link. Subsequent Figure S1 shows dropout rates at different study completion and data preparation points. Accordingly, 146 participants remained for analysis. The sample is described in Table 1.

**Table 1:** Demographical data of the participating female athletes (Mean and SD) and descriptive statistics of the questionnaires used in this study

Type of sports	Lean (*N* = 79)	Non-Lean (*N* = 67)	*p*
**Age (*Mean***,*** range***,*** SD***)	25.92 (18–65, 9.67*^1^)	22.88 (18–55, 5.01*^2^)	0.018*
**Meditation practice Min. per Month (*Mean***,*** SD***)	22.58 (68.48) [*N* = 78]^1^	14.96 (42.90)	0.417
**Main sports (*Mean***,*** SD***)			
Years of experience	14.27 (6.00)	13.84 (6.17)	0.670
Hours of weekly practice	7.25 (11.40)	5.02 (3.26) [*N* = 65]^1^	0.100
**Sport competitions (** ***N)***			
Yes No	32 47	45 22	0.001**
**BMI (kg/m²)**	21.43 (2.21)	22.48 (2.50) [*N* = 66]^1^	0.008**
**EAT*(Mean***,*** SD)***	6.92 (9.87)	8.22 (8.91)	0.419
**MBSRQ**,** BASS*(Mean***,*** SD)***	3.65 (0.67)	3.47 (0.63)	0.101
**MBSRQ**,** appearance scale*(Mean***,*** SD)***	3.76 (0.74)	3.41 (0.85)	0.01*
**SCS positive*(Mean***,*** SD)*** **SCS negative*(Mean***,*** SD)***	3.27 (0.73) 3.01 (0.80)	2.93 (0.60) 3.20 (0.63)	0.003** 0.115
**Occupation**			
School Study Full time job Part time job Retired	2 60 8 8 1	1 59 6 1 -	0.133

Note. ^1^N was indicated for variables with missing values only. *BMI* Body Mass Index (kg/m²), *MBSRQ* Multidimensional Body-Self Relations Questionnaire, *SCS* Self-Compassion Scale, *EAT* Eating Attitudes Test. *p <.05, **p <.01, ***p <.001. *^1^ 85.7% were 30 years or younger, the other eleven women were 31, 36, 37,40, 42, 29, 51. 53, 54, 56, and 65 years old. *^2^ 96.9% were 28 years or younger, one woman was 38 and another 55 years

The allocation of the participants to lean and non-lean sports is described in Table 2. Of the lean sports, the most practiced sport types were gymnastics and dancing, of the non-lean sports, tennis or volleyball.

**Table 2:** Allocation of Sport Types to Lean and Non-Lean Categories (N= )

Lean	Non-Lean
High-jump (1), long jump, triple jump, middle- /long- distance running (9), heptathlon (1), decathlon, judo, karate (1), tea kwon do (1), dancing (26), cycling, BMX-cycling, mountain biking (1), orienteering, paddling, rowing, ski jump (1), swimming (6), gymnastics (11), dog racing, biathlon, nordic combined, cross-country skiing (1)	Alpine skiing (3), snowboard, snow cross, hammer, discus, hurdle, sprint (2), javelin (2), freestyle ski, golf, shooting (4), fencing, motocross (1), horse riding (3), chess, table tennis, tennis (17), basketball (3), soccer (9), handball (7), indoor bandy, ice hockey, volleyball (10), beach volleyball (2), sailing, surfing

Other = 24. Note: Adapted from “Dieting to win or to be thin? A study of dieting and disordered eating among adolescent elite athletes and non-athlete controls” by M. Martinsen, S. Bratland-Sanda, A.K. Eriksson, J. Sundgot-Borgen. British Journal of Sports Medicine, 44, p. 71. (10.1136/bjsm.2009.068668). Copyright 2010 by the BMJ Publishing Group Ltd and British Association of Sport and Exercise Medicine. Adapted with permission

The study was preregistered at https://osf.io/f5uzk.

### Material

#### Demographic questionnaire

The demographic questionnaire consisted of questions regarding sex (as assigned at birth), age, education, occupation, mother tongue, meditation experience (years and hours per week), self-reported height, and weight (body mass index was calculated by kg/m²). Meditation experience was surveyed to ensure no systematic group differences in meditation experience because dispositional mindfulness is related to body image dissatisfaction [43]. Besides, to assess the proficiency level of the athletes, questions were asked regarding the primary sport, the length of time they have been participating, the number of hours they practice weekly, whether they compete, and whether they earn money from it (see Table 1).

#### Eating disorder risk

The Eating Attitudes Test (EAT-26 [44]; German Version [45]) is a standardized and validated self-report measure of trends associated with eating disorder. The questionnaire consists of 26 items, which can be accumulated into three subscales: Dieting (13 items, for example: “I am terrified about being “overweight”), bulimia and food preoccupation (6 items, for example: “I find myself preoccupied with food.”), and oral control (7 items, for example: “I cut my food into small pieces.”). The questions are scored on a 6-point Likert-Scale, asking how often individuals observe a certain behavior by them (“always” = 3, “usually” = 2, “often” = 1, “sometimes” = 0, “rarely” = 0, “never” = 0). This study calculated Cronbach’s alpha and McDonald’s Omega in R (version 4.2.3 [46]) using the package *psych* [47]. Item 9 had to be excluded from the reliability analysis due to zero item variance. Without item 9, Cronbach’s alpha was α =.91, and McDonald’s Omega was Ω =.93. For further calculations, the sum score of the 26 items was calculated.

#### Assessment of body image

For the assessment of body image, the Multidimensional Body-Self Relations Questionnaire– Appearance Scale (MBSRQ-AS [48]; German version [49]) was applied. The appearance scale of the MBRSQ is composed of 34 items divided into five subscales relating to aspects of appearance in body image. In line with the study of Jansen et al., [16] two subscales were chosen: a) Appearance Evaluation (7 items, example: “My body is sexually appealing”) and Body Areas Satisfaction Scale (BASS) (9 items, example: “How dissatisfied or satisfied are you with your weight?”). The items are measured on a 5-point Likert scale with the rating categories agreement (1 = definitely disagree to 5 = definitely agree), satisfaction (1 = very dissatisfied to 5 = very satisfied), or frequency (1 = never to 5 = very often). In a study of female participants, Cronbach’s alpha ranged from α = 0.73 to α = 0.89 [50]. In this study, the overall Cronbach’s alpha was α = 0.72, McDonald’s Omega was Ω =. 92. Cronbach’s alpha of the subscale Appearance Evaluation was α = 0.90, and McDonald’s Omega was Ω =. 93, and for the Body Area Satisfaction Scale, the values were α = 0.81 and Ω =. 87.

**Fig. 1:**
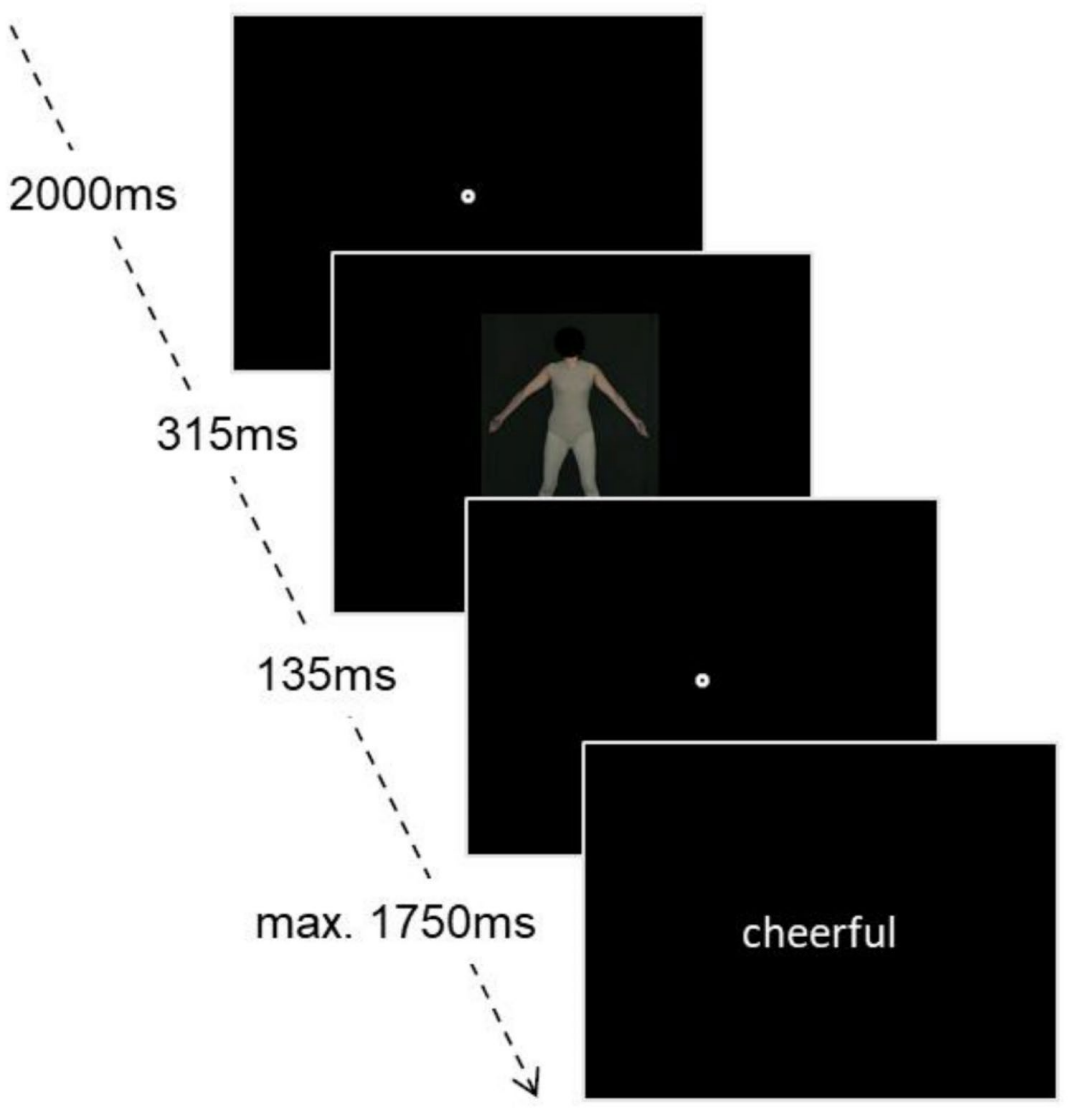
Implicit affective attitudes measurement

An additional measurement for body image satisfaction was the Photographic Figure Rating Scale [PFRS [4]; Austrian-version [51]; see an example picture in Fig. 1. The scale shows ten pictures of women wearing the same clothes whose body sizes correspond to different BMIs ranging from the lowest to the highest [4, 51]. Two pictures are summarized into one category, resulting in a total of five categories that are related to five BMI ranges: lowest (BMI < 15), low (BMI: 15-18.5), average (BMI: 18.5–24.9), high (BMI: 25.0-29.9), and highest (BMI ≥ 30). The participants were asked to select the most applicable image to each of the following questions: (1) the figure they found most attractive, (2) the figure with the biggest body size they still found attractive, (3) the figure with the thinnest body size, they still found attractive, (4) the figure they thought men would rate as most attractive (5) the figure that most reflected their own body size, and (6) the figure they considered to be the optimal body size.

To calculate the actual-ideal weight discrepancy, the ideal ratings (6) from current self-ratings (5) were subtracted. Both construct validity and test-retest reliability were at a reasonable level [4].

#### Self‑compassion

For the measurement of self-compassion, the Self-compassion scale was used (SCS [39]; German version [52]).

The SCS consists of 26 items rated on a 5-point Likert scale. The items refer to three positive subscales, such as self-kindness, common humanity, and mindfulness, and three negative subscales, such as self-judgment, isolation, and over-identification. For each of these scales, the mean score is calculated. In addition, two total scores of the negative and positive subscales can be calculated [53]. The 5-point Likert scale ranges from 1 =” almost never” to 5 =” almost always,” on which each item is rated. Total SCS scores evidenced good internal reliability with Cronbach’s Alpha of α = 0.92 and Mc Donald’s Omega of Ω = 0.93. Cronbach’s alpha for the positive was α = 0.89, and for the negative scale α = 0.87. Mc Donald’s Omega was Ω = 0.91 for the positive scale and Ω = 0.90 for the negative.

#### Explicit affective attitudes

Ten pictures from the Photographic Figure Rating Scale (PFRS) were chosen for the explicit rating task [4, 51]The images showed ten women wearing the same clothes and posture while their faces were covered. Five categories were built, which corresponded to five different BMI ranges (see the description of the PFRS above. For each of these images, three questions were asked regarding the attitude (“What is YOUR ATTITUDE towards the person in the photo?”), similarity (“How much do you LIKE the person in the photo?”) and closeness (“How CONNECTED/CLOSE do you feel to the person in the photo?”) the participant felt to the person pictured [54]. Questions were asked in randomized order, and the time given to answer was 5s. Items were rated on a 7-point Likert scale. Cronbach’s alpha and McDonald’s Omega were calculated for each category: Values for lowest BMI category were α = 0.80 and Ω = 0.88, for the low category α = 0.73 and Ω = 0.79, for the average category α = 0.72 and Ω = 0.84, for the high BMI category α = 0.77 and Ω = 0.87, and for the highest category α = 0.79 and Ω = 0.86. Following the paper by Hutcherson et al. [54] a composite score was calculated by the mean of the answers to the three questions.

#### Implicit affective attitudes

An affective priming task [54, 55] was applied to assess implicit attitudes using the same set of ten pictures from the PFRS. Before the experiment, participants underwent a brief practice exercise where they were presented with four photos of unfamiliar individuals with neutral expressions. First, a fixation point was shown (2000ms), followed by an image of a woman (315ms), followed by another fixation point (135ms). Finally, a randomly selected word was chosen from a pool of ten negative and ten positive words (shown for a maximum of 1750ms), selected from the Berlin Affective Word List (BAWL-R) [56]. Participants should indicate whether they rate the word as negative or positive by pressing the arrow keys. A total of 10 words with positive connotations (for example “honest”, “sunny”) and 10 words with negative (for example “unfair”, “sad”) connotations were presented, and each word was combined with each image once, resulting in a total of 20 trials. The trial was repeated if there was no response in the given time. Participants made, on average, 2.07 errors over the whole task (*SD* = 2.69). Participants had on average, 10.35% error trials, 0.35% trials with reaction times below 100ms, and 4.75% outliers with 2 *SD* above or below the group mean. These values were imputed by the mean of the correct trials in the respective group. Then, a composite score was built by subtracting the mean reaction time in the positive from the mean in the negative trials, see Fig. 1.

### Procedure

The experiment lasted about 20 min and was implemented as an online study using the programs OpenSesame and Survey JS on JATOS.org [57]. After being informed about the study and asked to provide their consent for participation, demographical data, body satisfaction, self-compassion, and eating disorder risk were surveyed. Subsequently, the PFRS and the explicit and implicit tasks were conducted, all following the order mentioned in this section. Participants were asked to use their laptop or computer for the appropriate completion of the experiment, as it did not work on mobile phones or tablets. Besides, only Chrome and Safari Browsers were allowed.

### Statistical analysis

To determine whether explicit ratings of pictures of women with different body sizes varied, a repeated measures ANOVA with the within-subjects factor “picture category” (pictures of individuals whose body size ranges from the lowest to the highest) and the between-subjects factor “group” (lean and non-lean sports) (Hypothesis 1) was conducted. Next, the study examined the potential difference in implicit affective ratings of the abovementioned pictures. A repeated measures ANOVA on the difference scores (reaction time) between negative and positive words, including the within factor “picture category” (pictures of individuals whose body size ranges from the lowest to the highest) and between-subjects factor “group” (lean sport, non-lean sport), was calculated. If sphericity was violated, data was corrected using either Greenhouse-Geiser or Huynh-Feldt corrections depending on the level of ε. The pairwise comparisons were Bonferroni corrected (p <. 01). The division into lean and non-lean sports was conducted according to the differentiation by Martinsen et al. [1] (see Table 2).

A power analysis with G*power [58] for the repeated measures ANOVA (within-between interaction) with a medium effect size *f* = 0.25, an alpha level of *p* =.05, and a power of 1-*ß* = 0.90 was conducted. *N* = 26 participants were required to test the two athlete groups’ explicit and implicit attitudes towards five different body size categories.

After this, two linear multiple regression analyses with the dependent variables BASS and appearance evaluation with the predictors own BMI, the actual-ideal weight discrepancy, the positive and negative scales of self-compassion, eating disorder risk, group, participation in competitions, explicit and implicit attitudes towards the picture of the lowest BMI category, and the interaction between the group * explicit (implicit) attitude towards the picture of the lowest BMI category were done.

With a medium effect size *f* = 0.15, an alpha level of *p* =.05, a power of 1-*ß* = 0.90, and 11 possible predictors, a power analysis for the linear regression resulted in a sample size of *N* = 152 women.

## Results

### Explicit and implicit affective attitudes

**Fig. 2:**
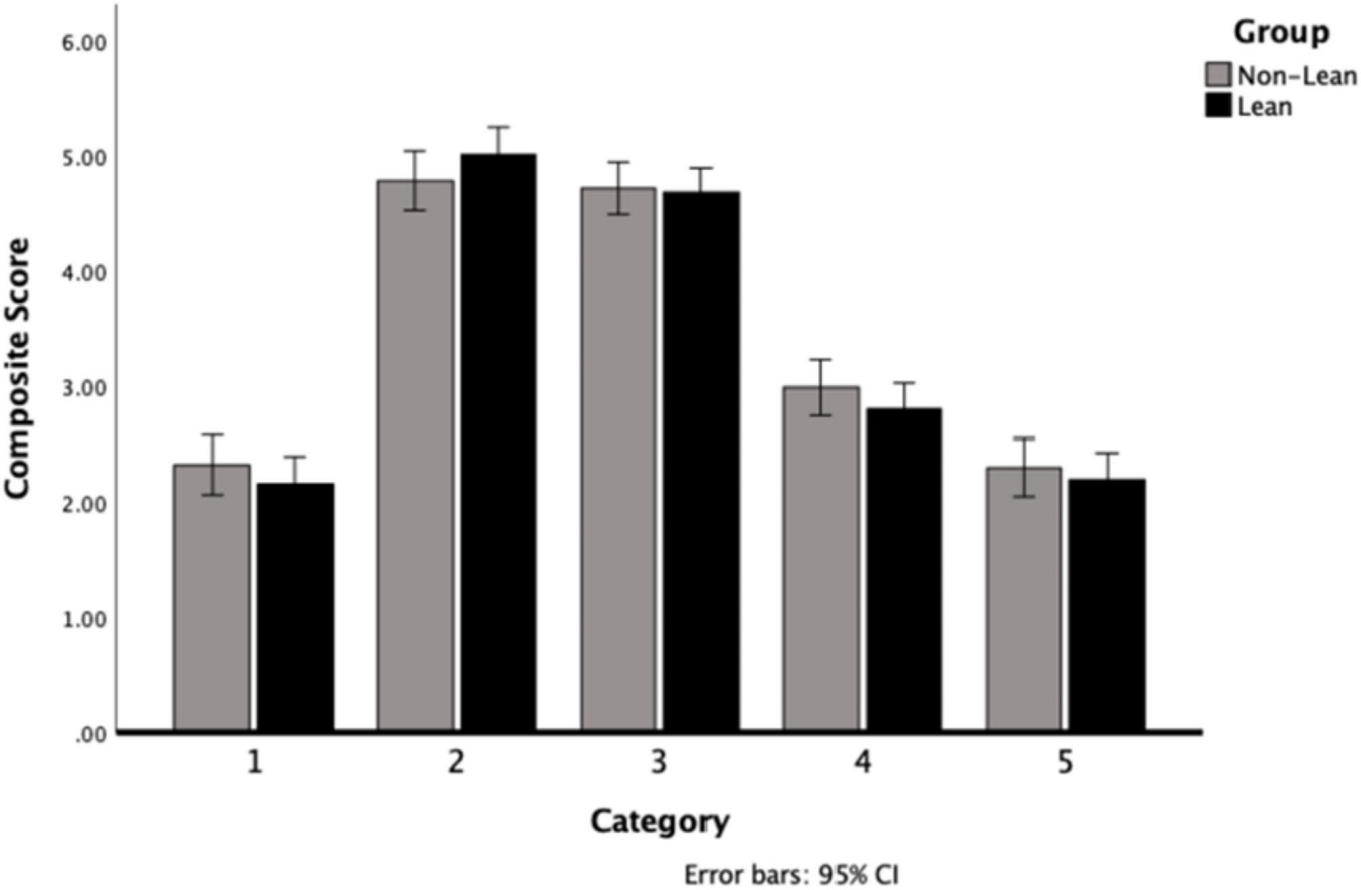
Measurement of Explicit Attitudes of Athletes. Note: Due to the problematic wording of the different pictures category in the original study, category was labelled 1–5; 1 = pictures of women whose body size corresponds to the lowest BMI; 2 = pictures of women whose body size corresponds to the low BMI; 3 = pictures of women whose body size corresponds to an average BMI; 4 = pictures of women whose body size corresponds to a high BMI; 5 = pictures of women whose body size corresponds to the highest BMI

Only the first part of Hypotheses 1 could be confirmed: The repeated measures ANOVA with the within-subjects factor “picture category” and the between-subjects factor “group” showed a significant main effect of “picture category” on explicit attitudes, *F*(2.68, 385.24) = 316.79, *p* <.001, $\:{\eta\:}_{\text{p}}^{2}$ = 0.69 (see Fig. 2). Pairwise comparisons showed that the images of the individuals whose body size corresponds to BMI ranges of the low and average category were rated significantly more positively than all other categories (*p* <.001), but not significantly from each other (*p* =.374). The main effect of “group” was not significant *F*(1, 144) = 0.23, *p* =.634, $\:{\eta\:}_{\text{p}}^{2}$ = 0.002, as well as the interaction of “picture category*group” *F*(2.68, 385.94) = 1.30, *p* =.277, $\:{\eta\:}_{\text{p}}^{2}$ = 0.009. Because both groups differ in their BMI, the BMI was considered exploratorily as a co-variate. This analysis was not preregistered. Only a significant interaction of “BMI*group” *F*(35.068, 412.566) = 12.07, *p* <.001, $\:{\eta\:}_{\text{p}}^{2}$ = 0.078. The attitude towards the pictures of the lower BMI category correlated negatively with the BMI, and the attitudes towards the higher and highest categories positively. In two other exploratory not preregistered analyses, the competition level was included as another independent variable, but there was no significant effect nor any interaction with group or picture category. In the second exploratory analysis, the number of participants was split into three groups: Group 1 included athletes from an aesthetic sport (including dancers and gymnasts), group 2 from a lean sport, and group 3 from a non-lean sport. There was no main effect of “group” nor an interaction of “picture category*group”.

Regarding the implicit reaction time difference, neither the main effect of “picture category” *F*(4,576) = 1.69, *p* =.150, $\:{\eta\:}_{\text{p}}^{2}$ = 0.01 nor the main effect “group” *F*(1,144)= 1.63, *p* =.204, $\:{\eta\:}_{\text{p}}^{2}$ = 0.01, nor the interaction of “picture category*group” *F*(4,576) = 0.93, *p* =.444, $\:{\eta\:}_{\text{p}}^{2}$ = 0.01 showed significant differences.

### Appearance evaluation and body areas satisfaction scale (BASS)

Two linear regression analyses were calculated for the Appearance Evaluation and the BASS of the MBSRQ. The assumptions for linear regression (linearity, normality, independence, and homoscedasticity) indicated multicollinearity in the interaction of the explicit attitude toward the lowest BMI category*group and the implicit attitude toward the lowest BMI category*group, so they were excluded from the analysis.

The analyses for BASS demonstrated that 36.6% (*R* =.61) of the variance was predicted by the model, *F*(9, 129) = 8.27, *p* <.001. The predictors of positive self-compassion (positively), actual-ideal weight discrepancy (negatively), and eating disorder risk (negatively) were significant (see Table 3).

**Table 3:** Regression-Analysis of body areas satisfaction

	Body area satisfaction							
Variable	*b*	*SE*	β	*t*	*p*	95% CI for *b* *LL UL*	Collinearity *Tolerance VIF*	
Intercept	3.63	0.73		4.94	< 0.001***	[2.18; 5.08]		
BMI	< 0.0.01	0.02	-0.01	-0.15	0.880	[-0.05; 0.04]	0.66	1.51
AIWD	-0.13	0.06	-0.23	-2.60	0.010*	[-0.29; -0.04]	0.62	1.63
Sport competition	0.03	0.10	0.02	0.30	0.763	[-0.68; 0.23]	0.87	1.16
Positive SCS	0.22	0.09	0.23	2.48	0.014**	[0.04; 0.39]	0.57	1.72
Negative SCS	-0.16	0.09	-0.18	-1.78	0.076	[-0.34; -0.02]	0.51	1.94
EAT	-0.02	0.06	-0.23	-2.61	0.010***	[-0.03; -0.01]	0.62	1.63
Group	-0.03	0.10	-0.01	-0.92	0.927	[-0.22; 0.20]	0.81	1.23
Explicit lowest BMI	0.07	0.05	0.01	0.13	0.896	[-0.09; 0.11]	0.86	1.15
Implicit lowest BMI	0.00	0.00	0.04	0.56	0.580	[0.000; 0.001]	0.91	1.10

*Note. BMI* Body Mass Index (kg/m²), *AIWD* Actual-ideal weight discrepancy, *SCS* Self-Compassion Scale, *EAT sum score* Eating Attitudes Test. *p <.05, **p <.01, ***p <.001

The model predicted 45.6% (*R* =.68) of the variance for the Appearance Evaluation, *F*(9, 130) = 12.12, *p* <.001. Three predictors, self-compassion (positively), the actual-ideal weight discrepancy (negatively), and eating disorder risk (negatively) were significant (see Table 4).

**Table 4:** Regression-Analysis of appearance evaluation

	Appearance Evaluation							
Variable	*b*	*SE*	β	*t*	*p*	95% CI for *b* *LL UL*	Collinearity *Tolerance VIF*	
Intercept	2.14	0.81		2.64	0.009***	[0.54; 3.73]		
BMI	0.04	0.03	0.13	1.60	0.111	[-0.01; 0.94]	0.67	1.49
AIWD	-0.30	0.06	-0.4	-4.96	< 0.001***	[-0.43; -0.18]	0.64	1.56
Sport competition	0.07	0.11	0.04	0.592	0.555	[-0.15; 0.28]	0.89	1.13
Positive SCS	0.30	0.10	0.26	3.10	0.002**	[0.11; 0.50]	0.58	1.72
Negative SCS	-0.07	0.10	-0.06	-0.65	0.519	[-0.27; 0.13]	0.51	1.94
EAT	-0.21	0.01	-0.25	-3.08	0.003**	[-0.35; -0.08]	0.63	1.59
Group	0.08	0.12	0.05	0.73	0.466	[-0.14; 0.31]	0.81	1.24
Explicit lowest BMI	-0.02	0.05	-0.02	0.28	0.781	[-0.12; 0.09]	0.89	1.12
Implicit lowest BMI	0.00	0.00	-0.08	-1.21	0.229	[-0.001; <0.000]	0.91	1.10

*Note. BMI* Body Mass Index (kg/m²), *AIWD* Actual Ideal Weight Discrepancy, *SCS* Self-Compassion Scale, *EAT sum score* Eating Attitudes Test. *p <.05, **p <.01, ***p <.001

Accordingly, hypothesis 2 was partly confirmed. However, attitudes did not predict the outcome variables significantly.

## Discussion

Regarding the first hypothesis, results have shown that explicit affective attitudes in female athletes are more positive towards pictures of women whose body sizes correspond to low and average BMI compared to images of women whose body sizes correspond to the lowest, high, and highest BMI. This supports the outcomes of the study of Jansen et al. [16]. No such effects regarding implicit attitudes could be found. Thus, the first hypothesis is only partly confirmed. Furthermore, no significant differences between the two sports categories (lean and non-lean) regarding their implicit affective attitudes toward the different picture categories were detected. It was not confirmed that athletes of a lean sport showed better implicit affective attitudes towards pictures of women whose body size corresponded to BMI ranges of the lowest to average BMI compared to high and highest BMI ranges. Lastly, explicit affective ratings in pictures of women whose body size corresponded to BMI ranges of low, average, and high were not ranked better among athletes in non-lean sports than athletes in lean sports. Turning to our second hypothesis, we found that the predictors for body area satisfaction and body appearance evaluation were actual weight discrepancy, self-compassion positive, and eating disorder risk.

### Implicit and explicit attitudes towards different body sizes in female athletes

First of all, the results in an athlete’s sample are comparable to those of a sample of non-athletes in terms of explicit and implicit attitudes [16]. The missing difference between the explicit and implicit attitudes of women in lean and non-lean sports might be due to the specific differentiation between lean and non-lean sports in this study that was orientated at the study of Martinsen et al. [1]. Learn sports were defined as sports in which leanness and/or low weight were considered necessary, and non-lean sports were defined as sports where these factors are considered less critical [1]. However, other sports groupings are shown in the literature, such as the division into technical, endurance, aesthetic, weight class, ball game, power, and antigravitation sports [22, 59]. Another classification into different types of sports in the present study, could have shown a clearer picture.

Furthermore, there was a difference in the number of lean athletes participating in competitions compared to non-lean athletes. This can indicate that women in lean sports focus less on performance, which can explain the non-existing differences between lean and non-lean athletes’ implicit affective attitudes towards different body sizes. This assumption is supported by a study by Varnes et al. [31], which shows that volleyball players on a higher competition level are more dissatisfied with their body size compared to lower competition levels and non-athletes. These findings could indicate that competition level and body image dissatisfaction are related; conversely, the results may not be specific to the outcome. Additionally, both groups differed in age. Eleven women were older than 30 in the non-lean sports group, whereas in the lean group, only two were. Some phases in the women’s lives (like pregnancy or menopause) might affect women’s bodies and body image. Only female athletes were included in this study. Perelman et al. [30] found that body dissatisfaction only appeared in male leanness athletes, not females. Accordingly, body image dissatisfaction in women may be caused by factors other than the sport performed, like the pressure from the media [60].

The finding that the explicit and implicit attitudes towards various pictures of women whose body size was related to the lowest BMI category were not associated with one’s body image satisfaction may be due to the choice of the lowest BMI category. It was chosen because we assumed that female athletes in lean sports show a higher positive affective attitude toward pictures of individuals whose body size corresponds to the lowest BMI ranges than athletes in non-lean sports. This part of hypothesis 1 was not confirmed for either category. Rerunning the regressions with the explicit and implicit attitudes towards various pictures of women whose body sizes were related to the low or average BMI range demonstrates a positive relation of the explicit attitudes towards pictures of women whose body size was related to the low BMI category. However, this analysis was not pre-registered but could hint that the attitudes towards pictures of women with a low BMI are relevant for aspects of body satisfaction. The result that some specific, explicit attitudes toward body image satisfaction are relevant provides further evidence for the need to investigate explicit attitudes toward different body sizes in more depth. Turning to our second hypothesis, we found that the predictors for body area satisfaction and body appearance evaluation were actual weight discrepancy, self-compassion positive, and eating disorder risk.

### Negative and protective factors related to body image satisfaction: the role of self-compassion and eating disorder risk

Eating disorder risk showed a negative relation to body image satisfaction, which aligns with other studies [34, 36]. (Former) elite women athletes recognized the relationship between eating disorder symptomatology and the sports environment in a way that the sports context increased or maintained their eating disorder symptomatology [61]. Contrary to the here presented results, Coelho et al. [62] found that lean athletes are at higher risk for eating disorders compared to non-lean athletes. A possible explanation can be the age of the participants in the present study. The group of non-lean athletes was, on average, 25.92 (9.67) years old. The critical age for the onset of eating disorders is in younger years [34]. Thus, the sample here might be too old to see significant differences between the groups.

Results showed that a higher level of self-compassion predicts less body image dissatisfaction. These findings are supported by another study of Wasylkiw [40]. Results in the present study indicated that the positive scale of SCS (self-kindness, common humanity mindfulness) predicts body area appearance and body area satisfaction. In contrast, the findings of Jansen et al. [16] hints the negative scale of SCS to be a predicting factor. The results that self-compassion was highly predictive of body satisfaction align with a recent meta-analysis of 59 studies (39 correlational studies and 20 intervention studies) investigating the relationship of self-compassion with eating and body image concerns in different samples [64]. They found a strong positive relation between a more positive body image and self-compassion, a medium negative relation between body-image concerns and self-compassion, and a medium negative relation between eating pathology and self-compassion. Turk and Waller [63] suggest that a potential mechanism is the relation of self-compassion with emotion regulation, as higher self-compassion allows the individual to apply adaptive coping mechanisms, like a mindful awareness and understanding of emotions, instead of avoiding negative emotions [64]. Maladaptive coping mechanisms may promote pathological eating behaviors, like binge eating or vomiting [63].

### Strengths and limitations

One strength of the study is that it investigates the relevance of implicit and explicit attitudes toward body size in female athletes. Both explicit and implicit measurements should be equally valuable methods for reaching a more holistic comprehension of attitudes toward specific aspects of various concepts, in this case, body image. This is important for changing behaviour, such as being more satisfied with one’s body size. For the implicit measurement, an affective priming paradigm was used; in further studies, the Standard Implicit Association Test (IAT) [65] can also be used.

One limitation is that the samples consisted mainly of recreational athletes doing their sports regularly but maybe at a lower level than elite athletes, which could be the most influential outcome factor. However, there was no relation of competition (yes/no) to the measurements of body image. Besides, the athletes were categorized into lean and non-lean according to the work of Martinsen et al. [1], but it is evident that athletes generally have low fat percentages. In a future study, the differentiation between sport types involves weight categories and those that do not include such categories. Also, most female athletes were young, which limits the generalisability of the findings for older female athletes. In future studies, a higher number of female athletes should be included. Furthermore, the PRFS images represent women whose body sizes correspond to BMI ranges, which differ from very low to very high. The photos do not depict an excessively athletic portrayal of the figure at any level (e.g., visible muscles). Also, the pictures of the PRFS were used to assess both body satisfaction and explicit and implicit attitudes. This ensured the comparability between the two measurements but also limited the results, as the measurement of body satisfaction may have primed the measurement of explicit and implicit attitudes. Due to the use of the PRFS, only female athletes could participate, making the results’ generalizability difficult.

Additionally, the explicit attitudes questions, even well investigated in former studies, might be challenging to answer regarding pictures of women without faces. The body image was not retrieved with a body image questionnaire specially designed for athletes, like the CBIQA [66, 67]. Neither the body’s ideal internalization nor the drive for thinness/muscularity was retrieved, which could be investigated in further studies compared to a control group including non-athletes.

## Conclusion

Even though the study is limited by the factors mentioned above, it contributes knowledge about the implicit and explicit attitudes of female athletes in lean and non-lean sports and the predicting relations of self-compassion and eating disorder risk toward body image. Future studies should consider experimental designs that investigate, for example, the effects of a self-compassion intervention on body image satisfaction. This investigation would offer the possibility to conclude whether self-compassion positively influences body image or whether the relationship is reversed. This would also shed light on promising treatment or prevention options.

## Electronic supplementary material

Below is the link to the electronic supplementary material.

Supplementary Material 1

## Data Availability

Data can be retrieved from https://osf.io/f5uzk.

## Abbreviations

BAWL-R: Berliner affective word list
BASS: Body area satisfaction scale
BMI: Body mass index
CBIQA: Contextual body image questionnaire for athletes
EAT: Eating attitudes test
IAT: Implicit association test
MBSRQ: Multidimensional body-self relations questionnaire
PFRS: Photographic figure rating scale
SCS: Self-compassion scale
