# Supplementary figures and images for: Explicit and implicit affective attitudes of female athletes towards different body sizes
Source: BMC Psychol. 2025 Mar 14;13:251. doi: 10.1186/s40359-025-02567-6 (PMC11908016; doi:10.1186/s40359-025-02567-6)

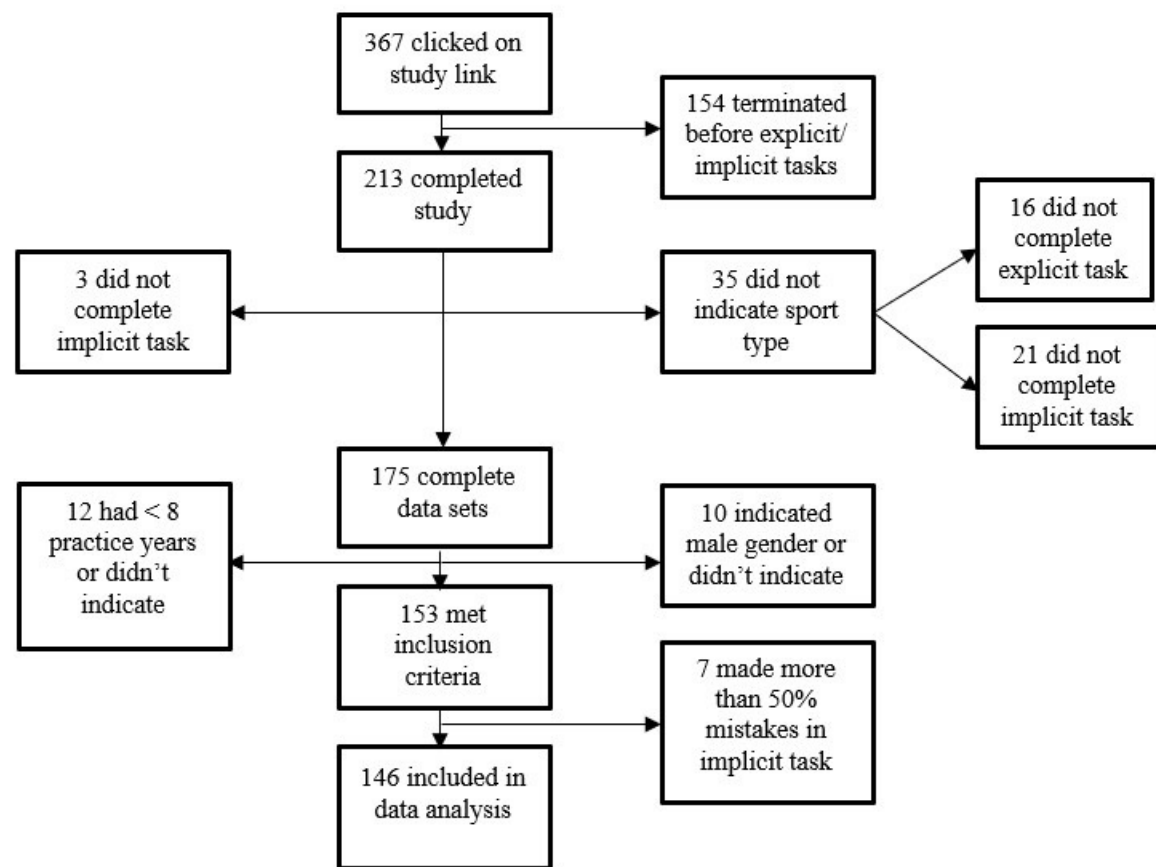

Supplement: Supplementary file 1 — Supplementary Material 1 [file 40359_2025_2567_MOESM1_ESM.pdf]
